# 

**Published:** 1852-09

**Authors:** 


*Books Received.*

WE have received from the publishers, Messrs. Blanchard & Lea, of Philadelphia, "Miller's Principles of Surgery," "Cooper's Lectures on Surgery," "Pirrie's System of Surgery," and "Meig's Obstetrics," all of which will be more fully noticed in the next number of the Journal.

We have also received the "Transactions of the Medical Society of the State of New York, Feb., 1852," "Transactions of the Medical Association of Southern Central New York, June, 1852," "Transactions of the Medical Association of the State of Missouri, April, 1852," and the "Transactions of the Twenty-ninth Annual Meeting of the Medical Society of Virginia, April, 1852."

*To our Readers.*

WE hope our friends will remember that we are dependent on *them*, in a great measure, for the interest we may be able to give our journal. We can think and write, at least we can *try*, but we cannot promise to *please* every one.

Short, practical articles—facts, such as our readers can use by the bed-side of their patients, will be especially acceptable. J.

### RECEIPTS

### FOR THE JOURNAL DURING THE MONTH OF AUGUST.

### Illinois.

| | |
|---|---|
| Dr. Thos. Stanton. - - - | $2 00 |
| " C. N. Andrews, - - - | 2 00 |
| " E. Winchister, - - - | 2 00 |
| " S. Shepard, - - - - | 2 00 |
| " G. W. Southwick, - - | 2 00 |
| " A W. Benton, - - - | 2 00 |
| " G. H. Watson, - - - | 2 00 |
| " J. C. Morfit, - - - - | 2 00 |
| " H. K. Palmer, - - - | 2 00 |
| " John Allen, - - - - | 2 00 |
| " J. A. Tyler, - - - | 2 00 |

### Indiana.

| | |
|---|---|
| Dr. S. R. Youngman, - - - | 2 00 |
| " Samuel Ross, - - - | 2 00 |
| " J D. Pew, - - - - | 4 00 |
| Drs C. D. & J. C Pearson, - | 2 00 |
| Dr. John Lewis. - - - - | 2 00 |
| " Jefferson Miller, - - | 2 00 |
| " Latta Wallace. - - - | 2 00 |
| " J. E. Heald, - - - | 2 00 |
| " M. Darnall, - - - - | 2 00 |
| Dr. Thos. D. Brown, - - | 2 00 |
| " J. B. Buchtell, - - - | 2 00 |
| " W. H. Gifford, - - - | 2 00 |

### Wisconsin.

| | |
|---|---|
| Dr. J. P. Hamilton, - - - | 2 00 |
| " E. R. Utter. - - - | 2 00 |
| " W. W. Blackman, - - - | 2 00 |
| " A. S. Castleman, - - | 2 00 |

### Michigan.

| | |
|---|---|
| Dr. L. W. Lovell, - - - | 2 00 |
| " M. Adams. - - - | 2 00 |
| " Jas. S. Skinner, - - - | 3 00 |

### Iowa.

| | |
|---|---|
| Dr. H. Fisk, - - - - | 2 00 |
| " H. C. Thurman, - - - | 2 00 |

